# Establishing a national knowledge translation and generation network in kidney disease: the CAnadian KidNey KNowledge TraNslation and GEneration NeTwork

**DOI:** 10.1186/2054-3581-1-2

**Published:** 2014-04-07

**Authors:** Braden Manns, Brendan Barrett, Michael Evans, Amit Garg, Brenda Hemmelgarn, Joanne Kappel, Scott Klarenbach, Francois Madore, Patrick Parfrey, Susan Samuel, Steven Soroka, Rita Suri, Marcello Tonelli, Ron Wald, Michael Walsh, Michael Zappitelli, for the CAnadian KidNey KNowledge TraNslation and GEneration NeTwork

**Affiliations:** Department of Medicine, University of Calgary, Calgary, Alberta Canada; Department of Community Health Sciences, University of Calgary, Calgary, Alberta Canada; Libin Cardiovascular Institute and Institute for Population Health, University of Calgary, Kragujevac, Alberta Canada; Department of Medicine, Memorial Medical School, St John’s, NL Canada; Family Medicine and Public Health, University of Toronto, Toronto, Canada; Division of Nephrology, Department of Medicine, University of Western Ontario, London, ON Canada; Department of Medicine, University of Saskatchewan, Saskatoon, SK Canada; Department of Medicine, University of Alberta, Edmonton, Alberta Canada; Department of Medicine, University of Montreal, Montreal, Quebec Canada; Division of Pediatric Nephrology, Department of Pediatrics, Faculty of Medicine, University of Calgary, Calgary, Alberta Canada; Alberta Children’s Hospital Research Institute for Child and Maternal Health, Calgary, Alberta Canada; Department of Medicine, Dalhousie University, Halifax, Nova Scotia Canada; Medicine, Centre de Recherche du Centre Hospitalier de l`Université de Montréal, Montréal, QC Canada; Division of Nephrology, St. Michael’s Hospital and the University of Toronto, Toronto, ON Canada; Li Ka Shing Knowledge Institute of St. Michael’s Hospital, Toronto, ON Canada; Division of Nephrology, McMaster University, Hamilton, Ontario Canada; Department of Pediatrics, McGill University Health Centre, Montreal, QC Canada

**Keywords:** Kidney disease, Knowledge translation, Clinical trials

## Abstract

Patients with chronic kidney disease (CKD) do not always receive care consistent with guidelines, in part due to complexities in CKD management, lack of randomized trial data to inform care, and a failure to disseminate best practice. At a 2007 conference of key Canadian stakeholders in kidney disease, attendees noted that the impact of Canadian Society of Nephrology (CSN) guidelines was attenuated given limited formal linkages between the CSN Clinical Practice Guidelines Group, kidney researchers, decision makers and knowledge users, and that further knowledge was required to guide care in patients with kidney disease. The idea for the Canadian Kidney Knowledge Translation and Generation Network (CANN-NET) developed from this meeting. CANN-NET is a pan-Canadian network established in partnership with CSN, the Kidney Foundation of Canada and other professional societies to improve the care and outcomes of patients with and at risk for kidney disease. The initial priority areas for knowledge translation include improving optimal timing of dialysis initiation, and increasing the appropriate use of home dialysis. Given the urgent need for new knowledge, CANN-NET has also brought together a national group of experienced Canadian researchers to address knowledge gaps by encouraging and supporting multicentre randomized trials in priority areas, including management of cardiovascular disease in patients with kidney failure.

## Résumé

Les patients atteints d’insuffisance rénale chronique (IRC) ne reçoivent pas toujours les soins recommandés dans les lignes directrices en raison de la complexité du traitement de l’IRC, de l’absence de données probantes sur différents aspects de l’IRC et d’un manque de diffusion des bonnes pratiques. Lors d’une conférence tenue en 2007 et réunissant différents acteurs clés impliqués en néphrologie, les participants ont attribué le peu d’impact des lignes directrices de la Société canadienne de néphrologie (SCN) à un manque de communication et d’arrimage entre le comité chargé des lignes directrices cliniques de la SCN et les chercheurs, les décideurs et les utilisateurs de connaissance. De plus, les participants ont aussi mentionné la nécessité d’acquérir plus de connaissances afin d’optimiser les soins de patients atteints d’IRC. L’idée du Canadian Kidney Knowledge Translation and Generation Network (CANN-NET) s’est développée suite à cette conférence. CANN-NET est un réseau pancanadien réunissant la SCN, la Fondation canadienne du rein et différentes sociétés savantes professionnelles et dont la visée est d’améliorer les soins et la survie des patients atteints ou à risque de maladie rénale. La détermination du moment optimal d’initiation de la dialyse ainsi que l’utilisation accrue des modalités de dialyse à domicile font partie des priorités initiales de transfert de connaissance identifiées par CANN-NET. De plus, compte tenu d’un besoin continu de nouvelles connaissances, CANN-NET a aussi créé un groupe national de chercheurs canadiens afin d’accroître les connaissances et l’acquisition de données en encourageant et en supportant des études cliniques randomisées et multicentriques sur des sujets jugés prioritaires tels que le traitement de la maladie cardiovasculaire chez les patients atteints de maladie rénale.

## Background to the need for the Canadian Kidney Knowledge Translation and Generation Network

Chronic kidney disease (CKD) is common [1, 2] and often coexists with, or is a complication of, diabetes, hypertension, and vascular disease [3]. Management of patients with CKD is complicated by the lack of randomized trial data to inform clinical care [4]. Even where good quality data from trials exist, results are often not translated into practice. This results in care of CKD patients that is not consistent with guidelines [5–7].

In 2008, the Canadian Society of Nephrology (CSN) published clinical practice guidelines for the care of patients with kidney disease [1]. The guidelines addressed the many diverse aspects of care for these patients, including management of hypertension and diabetes, anemia, and abnormalities of mineral metabolism. The guidelines identified many areas where further knowledge was required to guide care, and their impact may have been limited by the lack of formal linkages between the CSN Clinical Practice Guideline Group, kidney researchers, decision makers and knowledge users such as primary care physicians, regional renal programs, the Kidney Foundation of Canada, and patients themselves.

Recognizing the lack of capacity in knowledge translation and randomized trials [4] in kidney disease, two Canadian conferences: Horizons 2000 and Horizons 2015 were held to develop a Canadian strategic research agenda. The Horizons conferences brought together key stakeholders from the kidney research community and organizations including the Kidney Foundation of Canada (KFOC), the CSN, the Canadian Society of Transplantation (CST), and the Canadian Institutes for Health Research (CIHR).

At the Horizons 2000 conference, participants agreed to develop a transdisciplinary research training program to enhance capacity for kidney research in Canada. The Kidney Research Scientist Core Education and National Training Program (KRESCENT) program (http://www.krescent.ca ) developed from this meeting as a unique research training program for kidney scientists and allied health professionals [8]. The Horizons 2015 conference (held in 2007), whose focus was on enhancing excellence and capacity in kidney research, prioritized two other initiatives, including creating a network of kidney researchers across Canada, and creating stronger links between kidney researchers and knowledge users.

**Figure 1:**
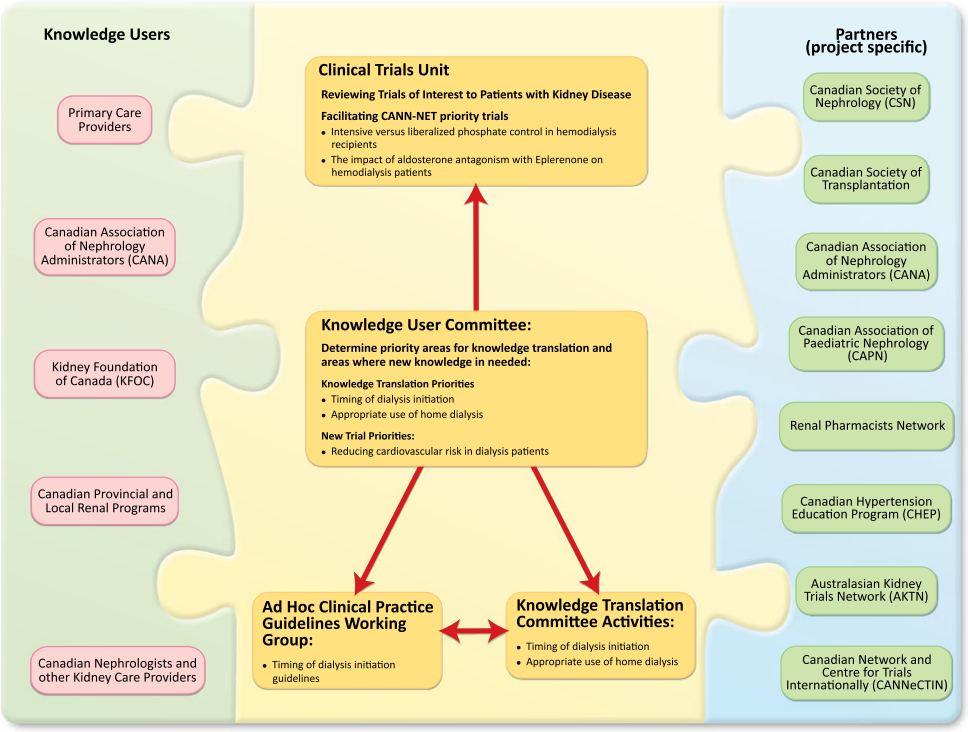
**CANN-NET Partners and Knowledge Users.**

The *Ca*nadian Kid*N*ey K*N*owledge Tra*N*slation and G*E*neration Ne*T*work (CANN-NET) was initiated in 2010 with funding from CIHR to address the Horizons 2015 priorities. CANN-NET developed linkages between Canadian kidney disease guideline producers, knowledge translation specialists and knowledge users to improve knowledge dissemination and the care of patients with kidney disease (Figure 1). CANN-NET has also brought together a national group of experienced Canadian researchers to address knowledge gaps by coordinating and executing multicentre clinical trials to address the need for new knowledge. To ensure that CANN-NET is relevant and responsive to knowledge user needs, CANN-NET partnered with key knowledge users from the kidney community, primary care, other partners with a vested interest in kidney health, experts in knowledge translation and global clinical trials.

## CANN-NET objectives

The overall goal of CANN-NET is to improve the care and outcomes of patients with kidney disease. This will be achieved by 1) linking an active clinical practice guidelines committee with relevant knowledge users, ensuring best practice for patients with CKD through knowledge dissemination of important guidelines to knowledge users, and 2) identifying knowledge gaps in kidney disease that can be addressed through large studies facilitated by a collaborative network of nephrology researchers.

## How CANN-NET developed to fulfill these objectives

**Figure 2:**
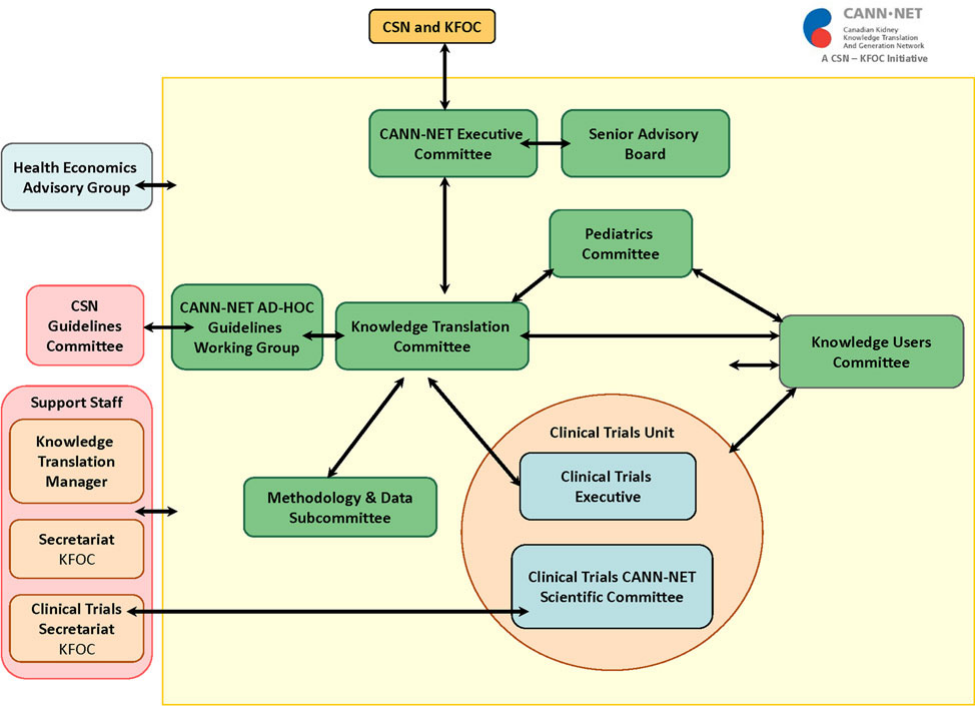
**CANN-NET Organizational Chart.**

To meet its diverse objectives, as illustrated in Figure 2, CANN-NET is organized into several working committees, including the knowledge user committee, the knowledge translation committee, the Pediatrics committee, and the Clinical Trials Scientific Committee. CANN-NET also has a close working relationship with the CSN guidelines committee. Each committee meets on a regular basis through video or tele-conference, and biannual face-to-face meetings. Terms of reference are available for each committee on http://www.CANN-NET.ca; the committee purpose, as well as duties and responsibilities are presented in Table 1.

**Table 1:** **Terms of reference for CANN-NET committees**

CANN-NET Committee	Purpose	Duties and responsibilities
CANN-NET Knowledge User Committee	To work with the Executive Committee and all of the CANN-NET working committees to guide CANN-NET priorities for new clinical trials and knowledge translation activity, and to facilitate knowledge translation activities	• To work in partnership with the Guidelines Committee to establish priorities for the new CSN guidelines
		• To work in partnership with the Knowledge Translation Committee to establish priorities for new Knowledge Translation Activities,
		• To facilitate creation of a network of key medical and administrative leads of renal programs and multidisciplinary CKD programs across Canada
		• To work in partnership with the Clinical Trials Group to provide feedback on priority areas for new knowledge
		• To ensure adequate communication between knowledge users, and the five working committees
CANN-NET Knowledge Translation Committee	To define, develop, monitor and evaluate knowledge translation activities	• To facilitate the dissemination and uptake of best practices for the care of patients with or at risk of kidney disease across Canada.
		• To develop a knowledge translation plan, using the knowledge to action cycle, to address priority areas established by the knowledge user committee
		• To conduct research to determine the optimal methods of knowledge dissemination to enhance the care of patients with kidney disease
		• To develop a framework for the evaluation of knowledge translation activities, including information to collect at baseline and after completion of KT activity
		• To advise the Knowledge Users Committee in the area of knowledge translation
CANN-NET Pediatric Committee	To define, develop, monitor and evaluate knowledge creation and translation activities relevant for Canada.	• To facilitate the dissemination and uptake of best practices for the care of children with or at risk of kidney disease across Canada.
		• To develop knowledge translation tools relevant for Pediatric Nephrology
		• To develop a framework for the utilization of databases and registries in Pediatric relevant kidney research
		• To assist and guide in the design of clinical trials including those trials which address knowledge gaps and knowledge user priorities, ensuring a focus on patient centred outcomes
		• To develop a framework for the evaluation of knowledge translation activities
CANN-NET Clinical Trials Scientific Committee	To determine which submitted kidney clinical trials should receive CANN-NET support or endorsement based on their merits, including their feasibility and importance to Nephrology	• To review and evaluate clinical trial proposals
		• To recommend which trials should receive CANN-NET support or endorsement
		• To develop and implement clinical trial review criteria and mechanism
		• To assist and guide in the design of nephrology clinical trials including those trials which address the Knowledge User Group priorities, ensuring a focus on patient centered outcomes
		• To liaise with the CANN-NET Knowledge Users Group and CSN Clinical Practice Guidelines Committee to develop clinical trial priorities

### Clinical Practice Guidelines Committee

CANN-NET works closely with the CSN’s existing clinical practice guidelines committee [9] which collaborates with Kidney Disease: Improving Global Outcomes (KDIGO), an international group that develops nephrology clinical practice guidelines [10]. To increase efficiency, the CSN clinical practice guidelines committee does not replicate guidelines produced by KDIGO; instead it develops commentaries on KDIGO guidelines to increase the relevance of these guidelines to Canadian practice and to facilitate guideline implementation and knowledge translation (KT) activities. In CANN-NET priorities areas where existing guidelines are not available, CANN-NET has collaborated with the CSN guidelines committee to establish “ad hoc” guideline working groups.

### Knowledge User Committee

In Canada, the care of patients with kidney failure is generally organized and funded through geographically based kidney care programs with dyad medical and administrative lead structures. These decision makers are unique since they typically hold multiple roles within kidney care programs, serving as administrators, clinicians, researchers and policy makers. CANN-NET has established a committee of these knowledge users, with the primary goal being to inform CANN-NET priorities for new clinical trials and to prioritize and facilitate knowledge translation activity.

While optimizing care and outcomes in CKD requires knowledge dissemination and new research across many areas of care, it is clear that not all areas can be addressed simultaneously. A mechanism for prioritizing action that incorporates the perspectives of patients with kidney disease and kidney-relevant decision makers was developed, considering a number of dimensions including a) whether there is a high level of evidence that adherence to a guideline will improve clinical or economic outcomes, b) whether clinical practice related to a particular guideline is highly variable and c) whether it is feasible to change clinical practice to improve concordance with guidelines.

The knowledge user committee helps to frame the implications of CANN-NET activities in terms of health care system functioning and impact, assessed over different time horizons.

### Knowledge Translation (KT) Committee

CANN-NET’s approach to KT is grounded in integrated knowledge translation [11] and involves a range of key knowledge users and their organizations (Figures 1 and 2). The KT committee works with the knowledge user committee to identify key guideline messages, determines the KT activities (expanded below), and communicates key messages in a format that meets the needs of knowledge users.

**Figure 3:**
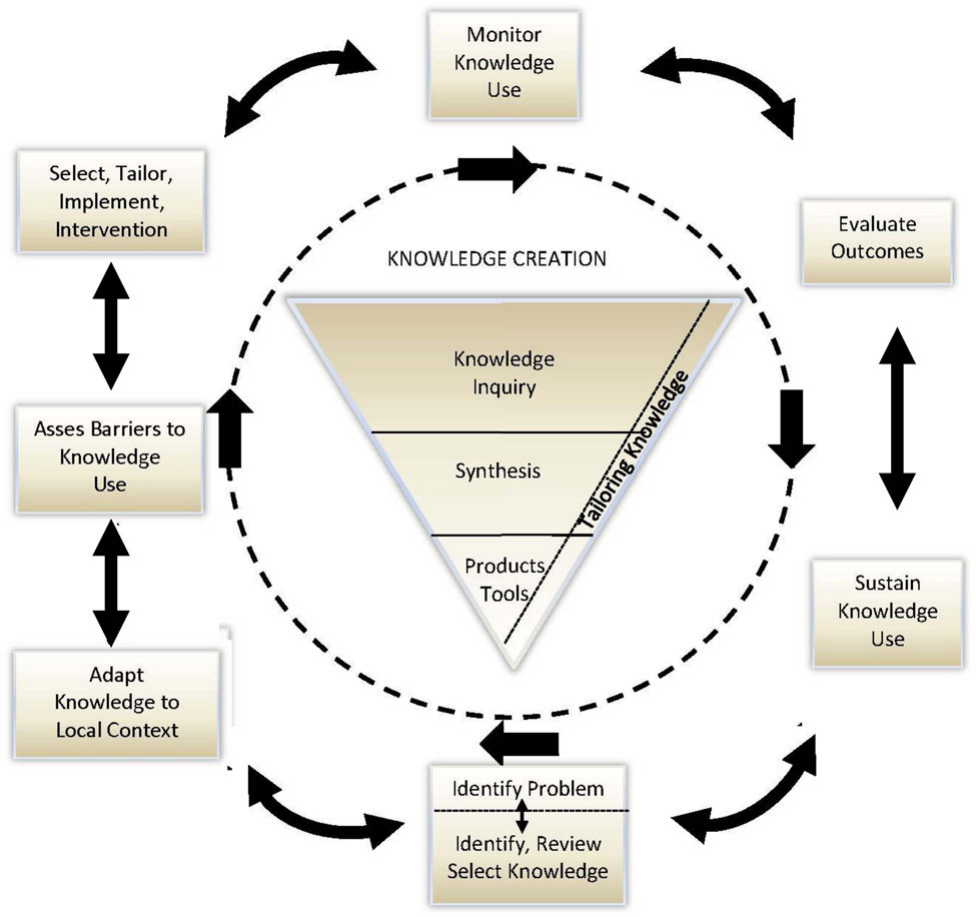
**Knowledge to Action Cycle.**

For each CANN-NET KT project, key activities are guided by the Knowledge to Action Cycle [12] (Figure 3) including the following steps:

- Examining barriers and facilitators using a multilevel approach that recognizes patient-, provider- and system-level factors
- Development of key themes and messages
- Selecting, tailoring and implementing interventions to address these barriers and facilitators, which may include:
- Writing a series of clinical and scientific summaries for various audiences
- Facilitating the annual CSN/Canadian Association of Nephrology Administrators symposium at the annual CSN meeting – an opportunity for dialog and education with renal program administrators from across Canada
- Creating key educational materials in priority areas, including infographics and animated white boards, aimed at patients and providers
- Working with local knowledge users including regional/provincial renal programs to develop tool kits to assist them in developing the local best strategy for getting the messages out
- Working in conjunction with the Canadian Organ Replacement Registry (CORR) to customize annual facility reports to include recommendations to increase achievement of quality care indicators in CANN-NET priority areas
- Monitoring knowledge use, and re-evaluating care and outcomes to determine if the KT activities are improving care and outcomes.

### Clinical Trials Committee

Since 2011, the Clinical Trials Committee has accepted requests for protocol review from nephrology researchers across Canada. It reviews the science, logistics, and feasibility of proposed trials using a transparent mechanism to identify the most promising studies, and critiques protocols to ensure that they are of the highest scientific standards. This critique is fed back to the investigators as constructive advice, in an iterative process if necessary, which the goal of improving the chances of the trials getting funded and proceeding to completion successfully. To facilitate multi-center trials, the Clinical Trials Committee is building a registry of research interests, expertise and resources across Canada. The committee is also interested in promoting the conduct of high quality trials that address priority areas but are initiated outside of Canada. Such central organization and support is critical to perform high quality clinical trials particularly in conditions affecting a limited number of people (e.g. kidney disease in children, vasculitis).

In 2010, the heads of renal programs were surveyed across Canada to determine the most important priorities for new clinical trials and two topics were identified by the Knowledge User committee as initial priorities. Although it was not intended that the Clinical Trials committee would plan trials from inception, members of the committee felt these initial priorities merited additional attention and resources. The top identified priorities were to determine the health impact of targeting different phosphate levels in patients on dialysis, and to determine the impact of aldosterone antagonism in reducing morbidity and mortality in dialysis patients. In collaboration with the Canadian Network and Centre for Trials Internationally (CANNeCTIN) and the Population Health Research Institute (PHRI) (two Canadian consortiums conducting trials investigating lowering cardiovascular risk), two pilot randomized trials addressing the feasibility of large trials to address these important clinical questions have begun (with one completing patient followup in November 2013).

### Pediatrics Committee

Current treatments for children with kidney disease are often supported by weak evidence or inferences made from clinical trials performed in adults. Therefore, in many management decisions, pediatric Nephrologists often have to use anecdotal or centre experience to guide therapy. To effectively conduct clinical research in children with kidney disease, multicentre trials and the expertise of researchers familiar with methodological challenges in performing clinical research in children are required. The Pediatrics Committee, which consists of members of the Canadian Association of Pediatric Nephrologists (CAPN), therefore has two main goals. The first goal is to identify areas specific to pediatric kidney disease which are in urgent need of evidence generation and knowledge translation to coordinate national and international efforts to achieve these goals. The second is to ensure that guidelines production and knowledge translation initiatives which are geared towards the care of adults with kidney disease, also consider the needs and are inclusive of children with kidney disease (for example: the timing of dialysis initiation, described below, was deemed relevant to child nephrology care and thus includes a pediatric-specific component). This is achieved by inclusion of Pediatrics Committee members in guideline commentary groups described above, membership of Pediatrics committee members in the Knowledge Translation and Clinical Trials committees and frequent communication between the Pediatrics committee and other relevant CANN-NET committee groups. This committee will also work with relevant knowledge partners to build a core group who can develop skills in clinical practice guideline productions, and knowledge translation relevant for children with kidney disease. Initial priority areas were identified by surveying CAPN membership, and include management of childhood nephrotic syndrome.

## CANN-NET partners and knowledge users: leveraging existing partnerships and building new ones

Partnerships are at the heart of all KT activity. Effective KT is dependent on effective bidirectional exchanges between researchers and knowledge users. To deliver on its KT mandate, CANN-NET will build on the strengths of others active in this field, create synergies, learn from existing experience and best practice, and avoid duplication of effort. Knowledge users play a key role in this initiative (Figure 1), and the Kidney Foundation of Canada is a funding partner and key knowledge user given its broad interest in optimizing care of patients with CKD. Other knowledge users include the leads of the provincial renal programs across Canada.

Given that CANN-NET cannot exist in isolation from other professional groups with complementary interests and scope, CANN-NET will also work closely with the following partners (Figure 1):

- Canadian Society of Nephrology (CSN), Canadian Society of Transplantation (CST), the Canadian Association of Pediatric Nephrologists (CAPN), and the Renal Pharmacists Network (RPN) – these are the professional societies of nephrologists and pharmacists in Canada caring for all patients with kidney disease, including adults, those with kidney transplants and children with kidney disease. Involvement of each will be critical since CANN-NET’s scope includes the continuum of patients with kidney disease and since guidelines (e.g. initiation of dialysis) and research will often be directly relevant to all societies.
- Canadian Hypertension Education Program (CHEP) – This collaboration will be critical where updates of guidelines include issues related to hypertension.
- Clinical trial networks including the Australasian Kidney Trials Network (AKTN) –a successful clinical trials network that has completed several high profile randomized trials in Nephrology [13] – and the Canadian Network and Centre for Trials Internationally (CANNeCTIN) – a national network funded by the CIHR/CFI Clinical Research Initiative program to improve the prevention and treatment of cardiac and vascular diseases and diabetes. The Population Health Research Institute (PHRI), which serves as the core Data Coordinating Centre for CANNeCTIN is also providing support to facilitate CANN-NET clinical trials of mutual relevance.

## What are the initial CANN-NET priorities, and why

Initial CANN-NET priorities for new clinical trials were developed based on knowledge user priorities – which as noted above related to improving management of cardiovascular disease in patients with kidney failure. Subsequently, CANN-NET has collaborated on a project that assessed the research priorities of patients on or nearing dialysis, their caregivers, and clinicians. The initial focus on patients on or nearing dialysis relates to the fact that quality of life of most dialysis patients is adversely affected, and considerable responsibilities are placed upon them and their caregivers. The method used was based on the approach successfully used in the UK by the James Lind Alliance (JLA) involving patients, their caregivers and clinicians in research priority setting. Initial results of this exercise are available at http://www.cann-net.ca/patient-information/research-priorities-survey#results and CANN-NET expects these to impact the types of research conducted and funded in the future.

Consistent with the Knowledge to Action Cycle [12], guideline and KT priorities were driven by an initial assessment of gaps in the care of patients with kidney disease. Knowledge gaps were identified using Canadian health care administrative data, including data from the Canadian Organ Replacement Registry (CORR). This was supplemented through a survey of knowledge users in 2010 to determine priorities for new clinical practice guidelines and KT activities. After development of an exhaustive list of topics for new guidelines and KT activities, topics were prioritized using the key dimensions noted above. Selection of the top priorities was finalized at a face-to-face meeting of knowledge users. Two priorities for KT were identified by knowledge users from adult renal programs including optimal timing of dialysis initiation, and increasing appropriate use of home dialysis, while one priority was identified by pediatric knowledge users, management of childhood nephrotic syndrome. With the recent release of the KDIGO guidelines for the care of patients with chronic kidney disease [14], it is expected that CANN-NET will develop additional priorities in nondialysis CKD.

## Timing of dialysis initiation

Over the past 10 years CKD patients in Canada are starting dialysis at higher levels of eGFR [15]. While consistent with existing guidelines [16], this practice is not consistent with recent information from a high quality clinical trial [13]. Given the potential for delaying administration of this very burdensome treatment and cost savings that might be realized if the new data were used to influence clinical care, knowledge users prioritized timing of dialysis initiation for guideline creation and knowledge translation activity. With respect to this initiative, each of the CANN-NET committee had predefined roles. The ad-hoc guidelines working group, established in collaboration with the CSN guidelines Committee, has completed clinical practice guidelines regarding the timing of dialysis initiation [17] The Knowledge User Committee has facilitated creation of an enduser network including medical and administrative leads of multidisciplinary CKD programs across Canada. These contacts will be critical for disseminating guidelines to the health care providers who can use them to optimize care, and have provided information about local practice that can guide KT activities.

The Knowledge Translation Committee used CORR data to establish current patterns of care with respect to timing of dialysis initiation across Canada, and surveyed providers to examine barriers and facilitators using a multilevel approach recognizing patient-, provider- and system-level issues [18]. After prioritization by the knowledge users, the key messages within clinical practice guidelines have been developed as the focus of KT efforts, directed towards the enduser network of CKD clinics. These messages will be incorporated into provider and patient directed infographics, animated whiteboards designed for patient education as well as academic detailing through continuing medical education – simultaneously targeting identified patient, and provider-level barriers. CANN-NET plans to evaluate the impact of these interventions on CKD clinics within a controlled trial using CORR data to measure outcomes.

## Increasing the appropriate use of home dialysis

The optimal dialysis modality blend has been reported to be 60-65% hemodialysis and 35-40% peritoneal dialysis. Home-based therapies (peritoneal and home hemodialysis) are under-utilized in many Canadian jurisdictions with the proportion of home-based therapies at 21.6% nationally and varying between 10 and 40% across centres [19]. In part, this is because decisions about dialysis modality selection involve a complicated interplay between health care provider knowledge and practice, patient and family values, patient autonomy, clinical benefits/limitations, resource allocation +/− incentives and geographical variation [20].

Several patient surveys have shown that patient preference should be the most important factor in dialysis modality selection, followed by comparative data on quality of life, morbidity, rehabilitation potential and survival [21–26] Other factors to consider are comorbid conditions, social circumstances and cost to patient/health system. Comparative studies suggest that quality of life, morbidity, rehabilitation potential and survival appear equivalent or possibly favour the use of home-based therapies, particularly in the initial years [27–32]. Despite this, home dialysis use remains low and variable across Canadian renal programs.

Based on this and the fact that home dialysis therapies are less resource intensive than in-center hemodialysis, increasing the appropriate use of home-based therapies has been identified as a priority by CANN-NET knowledge users. Since this issue is closely linked with timing of dialysis, knowledge users felt that a coordinated approach to both issues would be important. The framework for developing this approach will follow that identified above for timing of dialysis initiation.

## Childhood Nephrotic Syndrome

In 2012, the Kidney Disease Improving Global Outcomes (KDIGO) work group published Clinical Practice Guidelines for management of patients with Glomerulonephritis, which included guidelines for childhood nephrotic syndrome. To bridge the gap between available evidence and practice and identify how best to incorporate the KDIGO Guidelines into routine care, the Pediatrics Committee surveyed Canadian pediatric nephrologists to assess practice patterns in the management of childhood nephrotic syndrome. Survey results were compared with recommendations found in the Guidelines. They found potential evidence practice gaps in steroid dosing and duration, use of steroid-sparing agents and indications for kidney biopsy-the same areas there they noted high variability in practice. Since there is no knowledge translation strategy to implement the KDIGO Guidelines into pediatric nephrology in Canada, the Pediatrics Committee is developing an active knowledge translation plan for childhood nephrotic syndrome that includes development of a standardized national clinical pathway to promote uptake of evidence into practice.

## Moving forward: what is needed to optimize care and outcomes

Improving care and outcomes for patients with kidney disease cannot be done in isolation of clinicians, researchers, decision makers and patients. CANN-NET has received input from all groups, and will work with these groups to optimize care and outcomes for patients with kidney disease. Despite developing relationships with renal programs across Canada, further work to develop links with decision makers in provincial ministries of health will be required since some knowledge translation activities may require collaboration with government. For instance, managing patients with severe CKD not yet on dialysis is resource-intensive, but is remunerated at a lower rate than caring for dialysis patients. Aligning remuneration with important activities may assist appropriate care. Addressing other health-system barriers that might be identified may also require the collaboration of health ministries. Given that the initial CANN-NET initiatives, if successful, will improve patient-relevant outcomes and reduce health care costs, mutual collaboration with ministries would seem attractive.

## Summary

Through an iterative process, focused on improving the care of patients with kidney disease, CANN-NET has developed a robust collaborative framework which integrates clinical research, health outcomes evaluation, guideline generation and dissemination, and formal knowledge translation activities across Canada. With international and multidisciplinary collaborations, the potential impact of the initiative is far reaching. The initial activities and focus of CANN-NET on timing of dialysis initiation and dialysis modality selection, topics of key importance to patients and health care systems alike, is based on a systematic evaluation of needing to impact these two processes to improve outcomes. The potential for further generalization of lessons learned to other complex chronic conditions, is another potential value of CANN-NET to the health care community.

## Abbreviations

CKD: Chronic kidney disease
CSN: Canadian Society of Nephrology
CANN-NET: Canadian Kidney Knowledge Translation and Generation Network
KFOC: Kidney Foundation of Canada
CST: Canadian Society of Transplantation
CIHR: Canadian Institutes for Health Research
KRESCENT: Kidney Research Scientist Core Education and National Training Program
KDIGO: Kidney Disease: Improving Global Outcomes
KT: Knowledge Translation
CANNeCTIN: Canadian Network and Centre for Trials Internationally
PHRI: Population Health Research Institute
CAPN: Canadian Association of Pediatric Nephrologists
RPN: Renal Pharmacists Network
CHEP: Canadian Hypertension Education Program
AKTN: Australasian Kidney Trials Network
JLA: James Lind Alliance
CORR: Canadian Organ Replacement Registry.
